# Dynamically decreased miR-671-5p expression is associated with oncogenic transformation and radiochemoresistance in breast cancer

**DOI:** 10.1186/s13058-019-1173-5

**Published:** 2019-08-07

**Authors:** Xiaohui Tan, Zhongwu Li, Shuchang Ren, Katayoon Rezaei, Qing Pan, Andrew T. Goldstein, Charles J. Macri, Dengfeng Cao, Rachel F. Brem, Sidney W. Fu

**Affiliations:** 1 Department of Medicine, Division of Genomic Medicine, and Department of Microbiology, Immunology and Tropical Medicine, The George Washington University School of Medicine and Health Sciences, 2300 Eye Street, N.W. Ross Hall 402C, Washington, DC, 20037 USA; 2 Department of Pathology, Key Laboratory of Carcinogenesis and Translational Research (Ministry of Education), Peking University Cancer Hospital & Institute, Beijing, China; 3 Department of Pathology, The George Washington University School of Medicine and Health Sciences, Washington, DC, USA; 4 Department of Statistics, The George Washington University, Washington, DC, USA; 5 Department of Obstetrics and Gynecology, The George Washington University School of Medicine and Health Sciences, Washington, DC, USA; 6 Department of Radiology, The George Washington University School of Medicine and Health Sciences, Washington, DC, USA

**Keywords:** Breast cancer, 21T cell lines, miR-671-5p, EMT, Biomarkers

## Abstract

**Background:**

Understanding the molecular alterations associated with breast cancer (BC) progression may lead to more effective strategies for both prevention and management. The current model of BC progression suggests a linear, multistep process from normal epithelial to atypical ductal hyperplasia (ADH), to ductal carcinoma in situ (DCIS), and then invasive ductal carcinoma (IDC). Up to 20% ADH and 40% DCIS lesions progress to invasive BC if left untreated. Deciphering the molecular mechanisms during BC progression is therefore crucial to prevent over- or under-treatment. Our previous work demonstrated that miR-671-5p serves as a tumor suppressor by targeting Forkhead box protein M1 (FOXM1)-mediated epithelial-to-mesenchymal transition (EMT) in BC. Here, we aim to explore the role of miR-671-5p in the progression of BC oncogenic transformation and treatment.

**Methods:**

The 21T series cell lines, which were originally derived from the same patient with metastatic BC, including normal epithelia (H16N2), ADH (21PT), primary DCIS (21NT), and cells derived from pleural effusion of lung metastasis (21MT), and human BC specimens were used. Microdissection, miRNA transfection, dual-luciferase, radio- and chemosensitivity, and host-cell reactivation (HCR) assays were performed.

**Results:**

Expression of miR-671-5p displays a gradual dynamic decrease from ADH, to DCIS, and to IDC. Interestingly, the decreased expression of miR-671-5p detected in ADH coexisted with advanced lesions, such as DCIS and/or IDC (cADH), but not in simple ADH (sADH). Ectopic transfection of miR-671-5p significantly inhibited cell proliferation in 21NT (DCIS) and 21MT (IDC), but not in H16N2 (normal) and 21PT (ADH) cell lines. At the same time, the effect exhibited in time- and dose-dependent manner. Interestingly, miR-671-5p significantly suppressed invasion in 21PT, 21NT, and 21MT cell lines. Furthermore, miR-671-5p suppressed FOXM1-mediated EMT in all 21T cell lines. In addition, miR-671-5p sensitizes these cell lines to UV and chemotherapeutic exposure by reducing the DNA repair capability.

**Conclusions:**

miR-671-5p displays a dynamic decrease expression during the oncogenic transition of BC by suppressing FOXM1-mediated EMT and DNA repair. Therefore, miR-671-5p may serve as a novel biomarker for early BC detection as well as a therapeutic target for BC management.

**Electronic supplementary material:**

The online version of this article (10.1186/s13058-019-1173-5) contains supplementary material, which is available to authorized users.

## Introduction

Breast cancer (BC) represents one of the most significant disease burdens of any cancer worldwide. Tumor-free survival rate relies on early and accurate pathological diagnosis and appropriate treatment. Breast carcinogenesis assumes a gradual transition from normal breast epithelial cells to atypical ductal hyperplasia (ADH), to ductal carcinoma in situ (DCIS), and eventually to invasive ductal carcinoma (IDC) [1]. ADH is a proliferation of dysplastic, monotonous epithelial cell populations that include clonal subpopulations [2]. DCIS is an intraductal neoplastic proliferation of epithelial cells that is separated from the breast stroma by an intact layer of the basement membrane and myoepithelial cells [3]. Up to 20% ADH and 40% of DCIS lesions progress to invasive disease if untreated [2, 3]. Percutaneous core needle biopsy (CNB) following pathological analysis is the standard technique following an abnormal mammogram for diagnosis. However, CNB is less reliable in differentiating simple ADH (sADH) from ADH coexisted with DCIS and/or IDC (cADH), leading to misdiagnosis or unnecessary surgical excision. In addition, the management for patients with ADH and DCIS remains controversial. Most patients with sADH may only need a follow-up, whereas those cADH may undergo a conserving surgery followed by radiochemotherapy to reduce the risk of recurrence. However, radiochemoresistance is one of the major barriers to improving the free relapse and/or survival rate of patients [4–7]. Understanding the molecular mechanism during the stepwise progression of breast tumorigenesis is essential for identifying reliable biomarkers to prevent over- or under-treatment of patients diagnosed with ADH or DCIS.

Epithelial-to-mesenchymal transition (EMT) is a vital process to promote BC progression and chemoresistance [8]. The hallmark of EMT is the loss of E-cadherin expression and apical–basal cell polarity, accompanied by the gain of mesenchymal characteristics, including the acquisition of cell migration and invasion abilities, as well as an increased expression of mesenchymal markers, such as vimentin, fibronectin, and N-cadherin [9]. Up to 90% of human tumors originate from differentiated epithelium and are susceptible to EMT [10, 11]. Forkhead box protein M1 (FOXM1) is a transcription factor required for a wide spectrum of essential biological functions, including cell proliferation, cell cycle progression, cell renewal, cell differentiation, and tissue homeostasis. FOXM1 is an essential inducer of EMT to promote tumor progression and metastasis. In addition, FOXM1 contributes to drug resistance in breast cancer cells by enhancing DNA-damage repair pathways [12–16]. In our previous studies, we demonstrated that miR-671-5p inhibits proliferation and invasion by targeting FOXM1-mediated EMT and DNA repair in BC. In our present work, we extend our study to further demonstrate that miR-671-5p undergoes a dynamic change during the oncogenesis from ADH to DCIS to IDC in formalin-fixed paraffin-embedded (FFPE) tissue, blood, and a T21 series cell model that mimics specific stages of human BC progression. In addition, we demonstrated the role of miR-671-5p in inhibiting FOXM1-mediated EMT and DNA repair in every stage of the BC oncogenic transformation, as well as in cells under UV and chemotherapeutic agent treatment.

## Materials and methods

### Cell lines and cell culture

The 21T series cell lines were obtained as a kind gift from Dr. Vimla Band (University of Nebraska Medical Center). They were originally derived from the same patient with metastatic BC, including H16N2 (derived from normal epithelia), 21PT (derived from ADH), 21NT (derived from primary DCIS), and 21MT (derived from pleural effusion of lung metastasis). [17]. The cell lines were cultured in α-MEM supplemented with 10% fetal bovine serum, 2 mM l-glutamine (ThermoFisher Sci, USA), insulin (1 mg/ml), epidermal growth factor (12.5 ng/ml), hydrocortisone (2.8 mM), 10 mM 4-(2-hydroxyethyl)-1-piperazineethanesulfonic acid (HEPES), 1 mM sodium pyruvate, 0.1 mM non-essential amino acids, and 50 mg/mL gentamycin reagent (Sigma Chemical, USA) in a 37 °C humidified incubator with 5% CO_2_.

### FFPE samples

Tissue blocks were retrieved from the Department of Pathology at the George Washington University Hospital and Beijing Cancer Hospital at the Peking University School of Oncology. The blocks were subject to microdissection into the following components: normal, hyperplasia, DCIS, and IDC, as previously described [18]. The breast lesions were confirmed by pathological diagnosis following CNB and surgical excision. The blood samples were collected from 4 patients with benign breast lesions, 2 with ADHs, 6 with DCISs, and 1 with confirmed IDC diagnosis from the George Washington University Hospital with the IRB approval, and the informed consents were obtained from the participants.

### Microdissection, RNA extraction, and quantitative real-time reverse transcription-PCR (qRT-PCR)

Total RNA from FFPE samples and cell lines were isolated and quantitated as described previously [19]. Plasma was obtained as the cell-free supernatant remaining after centrifuging blood, collected in the presence of an anticoagulant. Total RNA was prepared and purified from a 300-μl plasma using miRNeasy Serum/Plasma Kit (Qiagen). miR-671-5p (Acc#: MIMAT00038800) expression was assayed using the Taqman MiRNA Reverse Transcription Kit (Applied Biosystems), and target gene expression (SYBR Green) was analyzed using the ABI 7300 System, as described previously [20].

### Dual luciferase reporter assay

Cells were plated (2 × 10^5^ cells/well) in 24-well plates and co-transfected with 100 ng of DNA with pEZX-FOXM1-3′UTR (wild type or mutant) expression clones inserted downstream of a secreted Gaussia luciferase (GLuc) reporter and 100 ng of DNA with pEZX-miR-671-5p or the pEZX-MT scrambled control (mock), using the FuGENE Transfection Reagent (Promega). Luciferase activities were determined with Secrete-PairTM Dual Luminescence Assay Kit (Genecopoeia). GLuc luciferase activities were normalized to SEAP luciferase expression for each sample.

### miRNA and plasmid transfection

miRNA transient-transfection was performed as described [19, 20]. Briefly, miRNA precursors (miR-671-5p mimic, inhibitors, and mock controls) were transiently transfected into each of the 21T series cell lines by Lipofectamine RNAiMAX (Life Technologies) using the Opti-MEM I Reduced Serum Medium (Life Technologies). Cells were subjected to further analysis after 24 h, 48 h, and 72 h post-transfection. For rescue experiments, the pcDNA3.1/FOXM1 plasmid containing full-length human FOXM1 cDNA without 3′UTR was a kind gift of Dr. Suyun Huang (MD Anderson Cancer Center). The T21 cell lines were co-transfected with miR-671-5p and pcDNA3.1-FOXM1 or pcDNA3.1 empty vector.

### Protein extraction and Western blotting

Cell lysates were prepared using the RIPA Buffer (ThermoFisher Sci) according to the manufacturer’s protocol, and Western blot analysis with chemiluminescent detection was performed using the ProteinSimple imaging system as described [21]. The following antibodies and dilution factors were used: FOXM1 rabbit polyclonal antibody (13147-1-AP, 1:800, Proteintech), anti-rabbit vimentin (5741, 1:200 Cell Signaling), anti-rabbit E-cadherin (3195, 1:400, Cell Signaling), anti-rabbit beta-actin (4970 s, 1:2000, Cell Signaling), anti-rabbit IgG conjugated to horseradish peroxidase (7074S, 1:2000, Cell Signaling), and anti-mouse IgG (7076S, 1:2,000, Cell Signaling).

### Immunofluorescence microscopy

Immunofluorescence assays were performed as described earlier [21, 22]. Briefly, 2 × 10^5^ cells were plated on glass coverslips in 6-well plates and allowed to settle overnight. Cells were fixed in 2% paraformaldehyde and then stained with primary and secondary antibodies. Confocal images were obtained using an LSM 510 Confocal Microscope (Carl Zeiss). The number of nuclei containing at least one localized area of immunofluorescence was determined by examination of the confocal images. Antibodies for immunofluorescence assays used were as follows: anti-rabbit vimentin (5741, 1:200 Cell Signaling), anti-rabbit E-cadherin (3195, 1:200, Cell Signaling), anti-BRCA1 (ab16780, 1:500, Abcam), Alexa Flour 568 goat anti-mouse IgG (1:500, Invitrogen), and Alexa Flour 568 goat anti-mouse IgG (1:500, Invitrogen).

### Matrigel invasion assays

Matrigel invasion assays were performed using the BD BioCoat™ Matrigel™ Invasion Chamber (BD Biosciences) as previously described [23]. Briefly, prior to the start of each experiment, 500 μl of warm (37 °C) serum-free DMEM medium was added to the upper and lower chambers and allowed to rehydrate for 2 h in a 37 °C cell culture incubator, while 8 × 10^4^ cells were transfected by either miR-671-5p mimic or mock control for 24 h and seeded onto the top chamber of pre-wetted inserts. Cells were incubated in a Matrigel chamber in a 37 °C humidified incubator with 5% CO_2_ for 24 h. The invasive cells present were fixed, stained with the Diff-Quick staining solution and counted (five microscope fields under the × 10 lens). Experiments were conducted twice and in duplicates for each cell line. Cell counts were performed on five non-overlapping random fields for each chamber, and four chambers were counted for each experimental point, with the percentage of invasive cells being normalized to corresponding controls.

### Radio-chemosensitivity and MTT assays

The cells transfected with miR-671-5p mimic, inhibitor, or their corresponding mock controls were washed with 1× PBS. The MTT (100 μl) working solution (5 mg/ml stock MTT diluted in opti-MEM to 0.5 mg/ml working solution) was added to each well and incubated at 37 °C with 5% CO_2_ for 3 h. The MTT solution was then removed, and 100 μl DMSO was added to each well and incubated in a 37 °C humidified incubator with 5% CO_2_ for 30 min. Color development was measured using a spectrophotometer at 570 nm on a plate reader (BIO-TEK Instruments) and quantified as per the manufacturer’s protocol (Promega, USA). For the radiosensitivity assay, the cells transfected with miR-671-5p mimic or inhibitor and their mock controls were plated on coverslips. The medium was replaced with PBS, and cells were irradiated at 20 J/m^2^ (the dose was measured using a UVX radiometer [UVP Inc., Upland, CA]) using a 254-nm UV-C lamp (UVP Inc., Upland, CA) through 3-mm-pore-size isopore/micropore polycarbonate filters (catalog number TSTP02500; Millipore), as described [24]. After irradiation, the previously removed medium was added back. MTT was added and absorbance was measured. For the chemosensitivity assay, the cells transfected with miR-671-5p mimic, inhibitor, or their corresponding mock controls were seeded in 96-well tissue culture plates. Cells were treated with various concentrations of cisplatin (20 μM), 5-fluorouracil (5-Fu, 5 μM), paclitaxel (10 μM), or epirubicin (100 nM). MTT was added, and absorbance was measured at different time points.

### Plasmid treatment and host cell reactivation (HCR) assays

pCMVLuc reporter gene plasmids (a kind gift from Dr. Kenneth H. Kraemer, National Cancer Institute, NIH) were dissolved in 10 mm Tris-HCl, 1 mm EDTA, pH 8 (TE buffer) to a final concentration of 100 μg/ml and poured in a petri dish to form 1D 2-mm thick layer. For UV treatment, the petri dish was placed on ice and irradiated by 1000 J/m^2^ of UV light. For the chemotherapeutic agents treatment, 1 μl aliquots of a stock solution of cisplatin (0, 5, 10, 20 μM), 5-Fu (0, 5, 25, 125 μM), epirubicin (200 nM), and paclitaxel (0, 50, 100, 200 μM) (Sigma-Aldrich, St. Louis, MO) in TE were added to 10 μg plasmid DNA dissolved in 200 μl TE buffer, and the samples were incubated at 37 °C for 6 h. At the end of the incubation period, 1 m NaCl was added to a final concentration of 0.2 m NaCl, and plasmid DNA was precipitated with 2 volumes of ethanol, extensively washed with 70% ethanol and dissolved in TE buffer. DNA repair capability was assessed using a host cell reactivation (HCR) assay with the pCMVLuc reporter gene plasmid treated by UV or chemotherapeutic agents [21]. Briefly, 4 μl (200 ng) of CsCl-purified pCMVLuc plasmids, damaged or undamaged, were co-transfected with miR-671-5p mimic, inhibitor, or their corresponding mock controls into 21T series cells using FuGENE® HD Transfection Reagent (Promega). For the rescue experiment, the pCMVLuc plasmids were co-transfected with miRNAs plus pcDNA3.1-FOXM1 plasmid. The luciferase activity was measured.

### Statistical analysis

miR-671-5p expression in clinical samples was analyzed by the exact two-sided binomial test. Data were expressed as mean ± standard error (S.E.). Permutation tests were performed for MTT assays between control and miR-671-5p mimic-transfected groups. The Student *t* test (two-tailed) was applied to Matrigel assay between the control and the miR-671-5p-transfected group. *P* values less than 0.05 were considered statistically significant.

## Results

### Expression of miR-671-5p decreased gradually in breast lesions during the BC oncogenic transformation

**Fig. 1:**
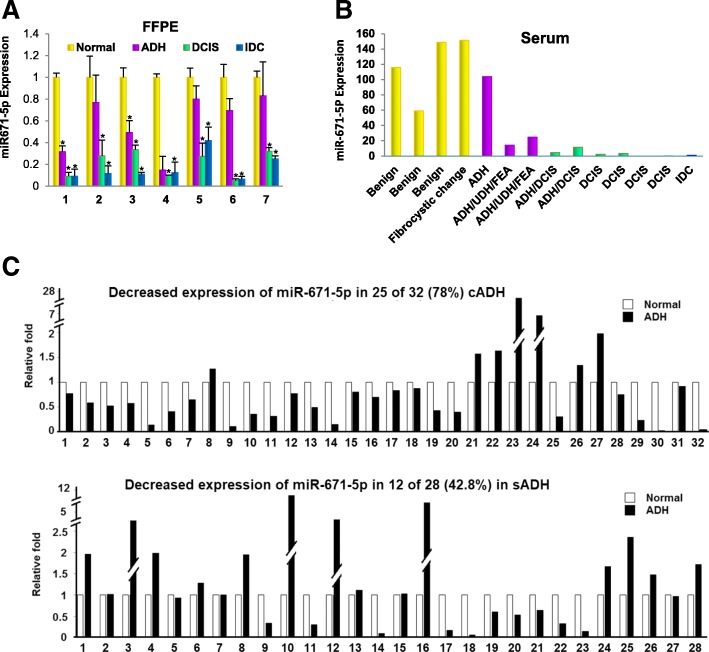
Expression of miR-671-5p in clinical samples during BC progression. **a** Expression of miR-671-5p was gradually downregulated in ADH, DCIS, and IDC compared to normal tissues in FFPE tissues. Seven FFPE tissues from each patient were microdissected into normal, ADH, DCIS, and IDC components before total RNA isolation and qRT-PCR analysis. Values represent the mean ± S.D. for three independent experiments (*p < 0.05 and **p < 0.01). **b** Expression of miR-671-5p in serum assayed by qRT-PCR. miR-671-5p expression was decreased in the serum of patients with ADH, DCIS, and IDC compared to that with benign breast lesions. **c** Differential miR-671-5p expression between sADHs and cADHs by qRT-PCR. Significantly decreased expression of miR-671-5p was presented in cADHs but not in sADHs, compared to their matched normal controls

In our previous work, we found decreased expression of miR-671-5p in BC compared to their adjacent normal tissues. We reasoned that miR-671-5p expression play an important role in BC oncogenic transformation. We firstly analyzed miR-671-5p expression in clinical samples undergoing the transition steps from ADH, DCIS to IDC in 7 FFPE BC tissues by isolating normal, ADH, DCIS to IDC components using microdissection technique. miR-671-5p expression was decreased gradually in ADH, DCIS, and IDC compared to normal tissues (Fig. 1a) in all seven cases. These results suggest that decreased expression of miR-671-5p is an early and gradual event during the progression of human BC.

Circulating miRNAs can be used as biomarkers for disease diagnosis, prognosis, and treatment. To determine whether the dynamic change of miR-671-5p can be readily detected in blood, we analyzed miR-671-5p expression by qRT-PCR in serum from patients of a separate cohort including 3 benign breast lesions, 1 fibrocystic breast, 3 ADHs, 6 DCISs, and 1 IDC. Consistent with the results from FFPE samples, miR-671-5p expression decreased gradually from benign breast lesions or fibrocystic breast to ADH, DCIS, and IDC (Fig. 1b). These results suggest that decreased miR-671-5p is an important risk factor in BC development and may serve as a biomarker detectable in both FFPE and serum.

### Decreased expression of miR-671-5p in cADH but not in sADH

Based on the clinical needs for the development of diagnostic markers to distinguish sADH and cADH, we next asked whether the miR-671-5p expression can distinguish between the two types of ADH. To address this, we examined the expression of miR-671-5p from a separate cohort. We found significantly decreased miR-671-5p expression in 25 of 32 (78%) cADHs compared with their matched adjacent normal controls (*p* < 0.01). In contrast to the cADHs group, the decreased miR-671-5p expression was only observed in 12 of 28 (42.85%) in sADHs. There is no statistically significant difference between sADH compared with their matched adjacent normal controls (Fig. 1c). However, there is a statistically significant difference between cADH and sADH groups (*p* < 0.001). This data suggests that aberrantly expressed miR-671-5p might be used to distinguish ADHs from ADHs.

### miR-671-5p directly targets FOXM1 in all 21T cell lines

**Fig. 2:**
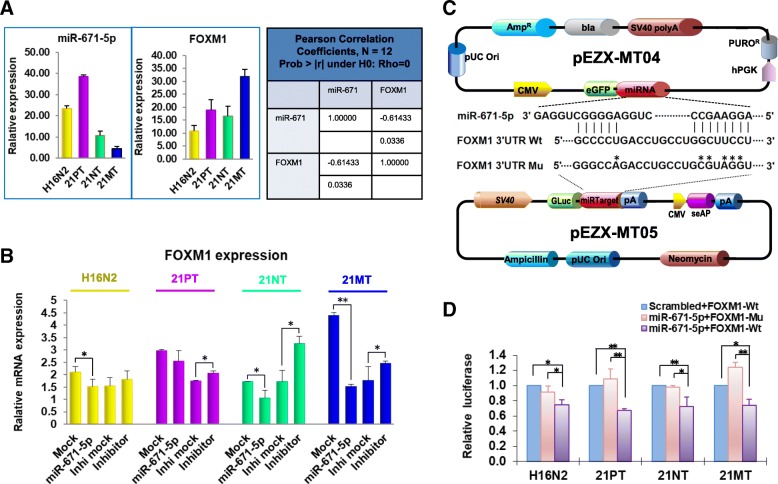
miR-671-5p directly targets FOXM1 in the 21T cell lines. **a** Inverse correlation between miR-671-5p and FOXM1 expression in 21T cell lines was detected by qRT-PCR. The left two panels represent qPCR results. The right panel show statistical analysis. The Pearson correlation coefficient between miR-671-5p expression and FOXM1 expression is − 0.61 with a *p* value of 0.0336, indicating a relatively strong and statistically significant negative relationship between miR-671-5p and FOXM1 expression. **b** FOXM1 expression was significantly repressed after miR-671-5p transfection in H16N2 cell line, and rescued by miR-671-5p inhibitor transfection in both H16N2 abd 21MT cell lines. **c** pEZX-MT05 vector was inserted with wild-type binding site in the 3′UTR of FOXM1 (FOXM1 3′UTR Wt) and the mutant sequence (FOXM1 3′UTR Mu) corresponding to miR-671-5p sequence that inserted into pEZX-MT04 vector. The mutated nucleotides were indicated by star symbols. **d** Relative luciferase activity was measured in 21T cell lines co-transfected with either 200 ng of pEZX-MT04-miR-671-5p or a scrambled control and 100 ng of pEZX-MT05-FOXM1-Wt or pEZX-MT05-FOXM1-Mu by FuGENE® HD Transfection Reagent (Life Technologies) for 48 h. The luciferase activity was significantly decreased in all 21T series cell lines when co-transfected with miR-671-5p and FOXM1-Wt. Values represent the mean ± S.D. for three independent experiments (*p < 0.05 and **p < 0.01)

We observed different effects of miR-671-5p on FOXM1 between triple-negative BC (TNBC) cell lines and non-triple negative ones [19]. We then attempted to determine whether miR-671-5p exerts its effect differently during BC oncogenesis. We employed the 21T cell lines, which were originally derived from the same patient with metastatic BC (Band reference?). These cell lines have been served to mimic specific distinct stages of human BC progression. The cell lines allowed us to monitor the dynamic changes of miR-671-5p through different stages during the oncogenic transition [17]. We found a significant inverse correlation between miR-671-5p and FOXM1 expression in the 21T cell lines (Fig. 2a). Forced expression of miR-671-5p significantly repressed FOXM1 expression in both mRNA and protein levels (Fig. 2b and Fig. 5c). Transfection of miR-671-5p inhibitor resulted in significantly increased FOXM1 expression. The repression of miR-671-5p is more significant in 21 NT and 21MT, suggesting that it affects FOXM1 expression in each step of BC progression, especially in DCIS and IDC stages. To confirm that miR-671-5p directly targets FOXM1 in BC progression, the luciferase reporter assay was performed. After co-transfection of the plasmids containing miR-671-5p and FOXM1 3′UTR wild-type sequence into the 21T cells, luciferase activity was significantly decreased in all four cell lines compared to the co-transfection of those containing either miR-671-5p plus FOXM1 3′UTR mutant sequence or scrambled control plus FOXM1 3′UTR wild-type sequence (Fig. 2c, d). Our data demonstrate that miR-671-5p specifically targets the 3′UTR of FOXM1 at 828–848 nt in all 21T cell lines, suggesting that miR-671-5p targets FOXM1 in all stages during BC transition.

### miR-671-5p inhibits FOXM1-mediated proliferation and invasion during the BC oncogenic transition

**Fig. 3:**
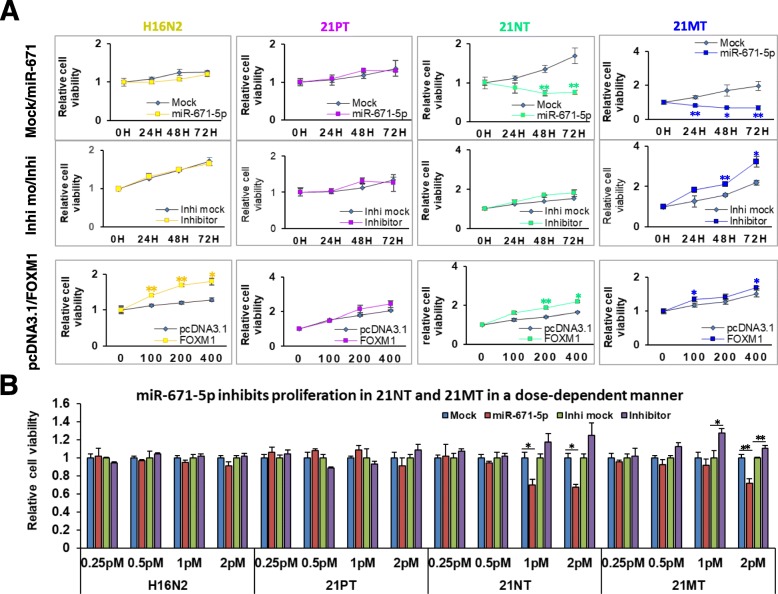
miR-671-5P inhibits proliferation in a time- and dose-dependent manner in the 21T cell lines. The 21T cell lines were transfected with mock control, miR-671-5p mimic, and inhibitor control and miR-671-5p inhibitor. **a** The proliferation rate was decreased after transfection of miR-671-5p mimic in 21NT and 21MT, while there were no significant changes in 21PT and H16N2 cells lines compared to the mock control (top panel). Inversely, transfection of miR-671-5p inhibitor significantly promoted the proliferation in 21MT while slightly in 21NT, but not in H16N2 and 21PT compared to the inhibitor mock (middle panel). The T21 cell lines were co-transfected with miR-671-5p mimic and pcDNA3.1-FOXM1 or pcDNA3.1 empty vector. **b** Forced expression of FOXM1 rescued miR-671-5p-mediated cell proliferation in a dose- and time-dependent manner. Values represent the mean ± S.D. for three independent experiments (*p < 0.05 and **p < 0.01)

We have previously demonstrated the tumor suppressor function of miR-671-5p in BC cell lines [19]. In this study, we focused on the role of miR-671-5p expression during breast oncogenesis. We firstly addressed the effect of miR-671-5p on cell proliferation. After ectopic expression of miR-671-5p, cell proliferation was significantly inhibited in 21NT and 21MT cell lines in a dose- and time-dependent manner, as compared to mock control. However, miR-671-5p overexpression did not show significant proliferative inhibition in H16N2 and 21PT cell lines (Fig. 3a, top panel). Conversely, transfection of miR-671-5p inhibitor significantly increased cell proliferation in 21MT cell lines, slightly in 21NT, but not in H16N2 and 21PT (Fig. 3a, middle panel). These results indicate an anti-proliferative effect of miR-671-5p, which is more significant in the advanced progression of BC. We then performed rescue experiments to further validate that FOXM1 targeting is involved in miR-671-5P-mediated proliferation inhibition in BC cells. Forced expression of FOXM1 not only abrogated the suppressive proliferation induced by miR-671-5p transfection in 21NT and 21MT cell lines, but also increased proliferation in 21PT and H16N2 cell lines (Fig. 3a, bottom panel).

**Fig. 4:**
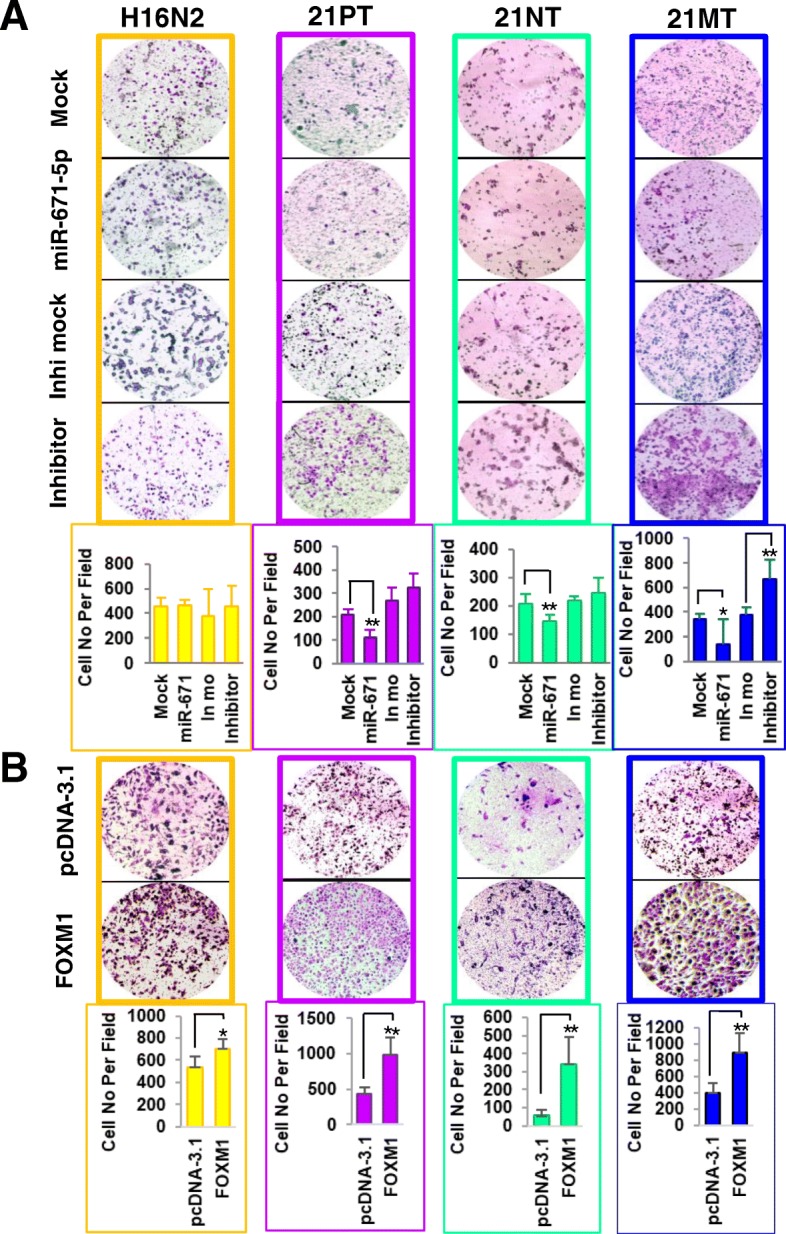
Effect of miR-671-5p on cell invasion. **a** Transwell Matrigel assays were performed for the invasion activity of 21T cell lines transfected with mock control, miR-671-5p mimic, inhibitor control, and miR-671-5p inhibitor. Overexpression of miR-671-5p significantly inhibits cell invasion in 21PT, 21NT, and 21MT cells, but slightly in H16N2 cells. **b** Transwell Matrigel assays showed that re-expression of FOXM1 abrogated invasion suppression by miR-671-5p in 21TP, 21NT and 21MT cells. Invasion ability of the cells was displayed as a percentage of the absolute cell numbers (right). Five fields of unit area on each membrane or whole membrane were counted for cell numbers, and the experiments were repeated three times with triplicates. Results are displayed as mean data ± S.D. (*p < 0.05)

We next tested the effect of miR-671-5p on cell invasion during the progression of BC using Transwell assays. miR-671-5p ectopic expression resulted in a significant inhibition of invasive capability in 21PT (46%), 21NT (30%), and 21MT (59%), but not in H16N2 cells when compared to the mock control. Conversely, transfection of miR-671-5p inhibitor significantly elevated cell invasion in 21MT cells, moderately in 21PT and 21NT (albeit not statistically significant) but not in H16N2 (Fig. 4a) when compared to the inhibitor mock. Rescue experiments by re-expression of FOXM1 not only abrogated the invasion suppression induced by miR-671-5p in 21TP, 21NT, and 21MT, but also increased cell invasion in H16N2 cells (Fig. 4b). These results suggest that miR-671-5p might suppress invasion by targeting FOXM1 in both precancerous lesions and invasive stages.

### miR-671-5p reverses the FOXM1-induced EMT in the 21T cell lines during the BC progression

**Fig. 5:**
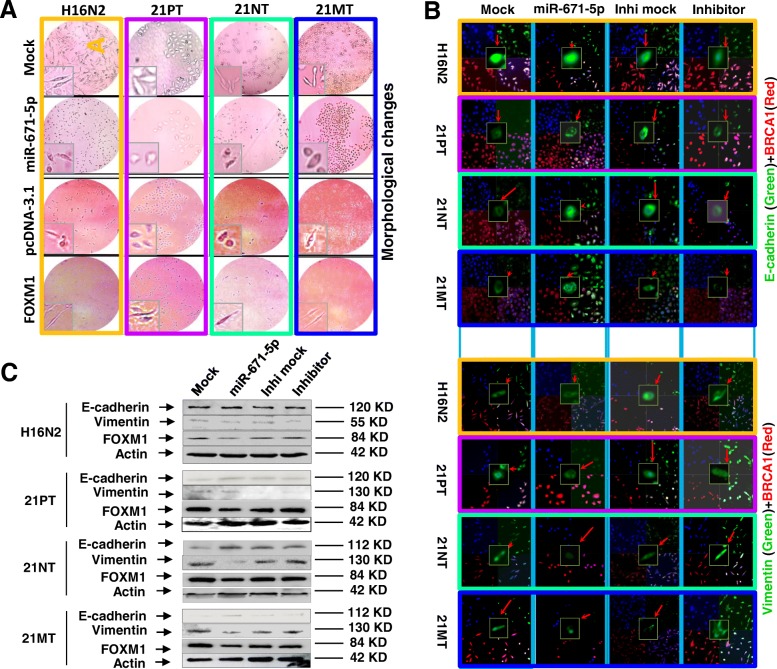
miR-671-5p reverses the FOXM1-induced EMT in the 21T cell lines. **a** Overexpression of miR-671-5p shifts the 21T cells from EMT to MET phenotype. Cell morphology was observed by microscopy in 21T cells 72 h after miRNA transfection. miR-671-5p-transfected cells showed a more epithelioid appearance compared to the mock-transfected cells, which showed elongated, irregular fibroblastoid shape (top panel). For rescue experiments (bottom panel), the pcDNA3.1/FOXM1 plasmid containing full-length human FOXM1 or pcDNA3.1 empty vector was transiently transfected 72 h after miRNA transfection. Cell morphological photos were recorded by microscopy after 72 h re-expression of pcDNA3.1 or pcDNA3.1/FOXM1 plasmid. FOXM1-transfected cells showed more elongated, irregular fibroblastoid morphology whereas pcDNA3.1-transfected one showed more MET state. EMT markers were analyzed by immunofluorescence staining (**b**) and Western blot (**c**) following the transfection of mock, miR-671-5p mimic, inhibitor mock, and miR-671-5p inhibitor in the indicated cells

In previously published work, we demonstrated that miR-671-5p reversed EMT to mesenchymal-to-epithelial transition (MET) phenotype in MDA-MB-231 BC cell lines by targeting FOXM1 [19]. We now aimed to determine whether miR-671-5p affects EMT during BC progression. The morphological change is an important parameter in EMT, widely used for assessing EMT. After 48 h and 96 h from the time of transfection, the morphology of cells was evaluated by microscopy. Significant morphological changes (from elongated, fibroblast-like spindle-shaped to round-shaped cells) were observed in miR-671-5p-transfected 21T series cells compared to the mock-transfected controls. Such observations indicate that the forced expression of miR-671-5p leads to the acquisition of MET phenotype in all 21T cells. Transfection of miR-671-5p inhibitor or re-expression of FOXM1 reversed the 21T cell lines from MET to EMTstage (Fig. 5a and Additional file 1: Figure S1). Consistent with the morphological changes, immunofluorescence and Western blot analyses revealed an upregulation of the epithelial marker E-cadherin as well as concomitant downregulation of the EMT marker, vimentin in miR-671-5p-transfected cells when compared to the mock control. Reversed expression of E-cadherin and vimentin was observed with the transfection of miR-671-5p inhibitor compared to that of the inhibitor mock. At the same time, FOXM1 was significantly downregulated in all miR-671-5p-transfected 21T cell lines compared to the mock controls (Fig. 5b, c). Rescue experiments by transfections of FOXM1 abrogated the effect of miR-671-5p on EMT alleviation. These findings suggest that miR-671-5p prevents oncogenesis by inhibiting FOXM1-mediated EMT.

### miR-671-5p sensitizes the 21T series cell lines to UVC exposure and chemotherapeutic agents

**Fig. 6:**
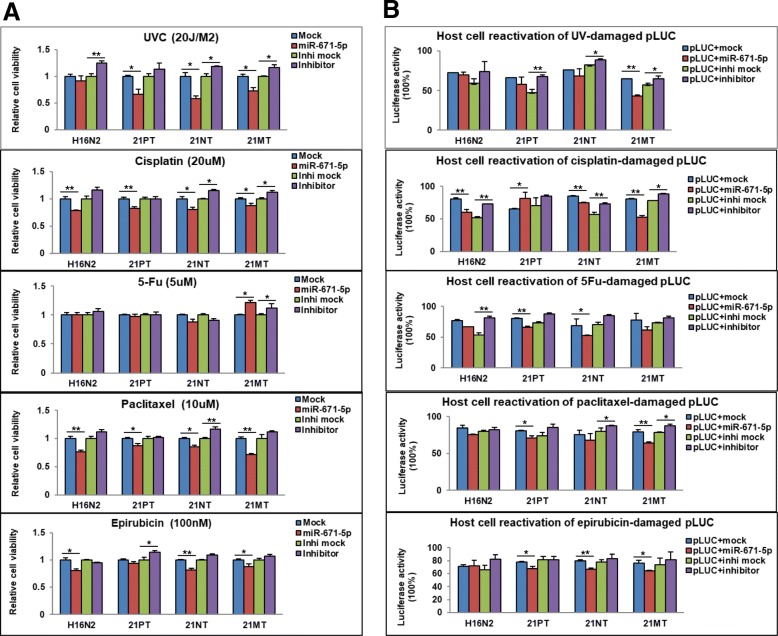
miR-671-5p sensitizes the 21T series cell lines to UVC exposure and chemotherapeutic agents by alleviating DNA repair capability. **a** The sensitivity of UV exposure and drug treatment was measured by MTT assay. The 21T cells were transfected with mock, miR-671-5p mimic, inhibitor mock, and miR-671-5p inhibitor. Cells were treated with UVC and chemotherapeutic agents 48 h after transfection. MTT was added, and absorbance was measured at different time points. miR-671-5p overexpression significantly increased sensitivity of 21T cells to UV, cisplatin, paclitaxel, and epirubicin but not to 5-Fu compared to the mock-transfected one. **b** HCR assay was performed to quantitatively measure DNA repair capability in 21T cells. Results are displayed as mean data ± SE. **p < 0.01, *p < 0.05

Radiation therapy or chemotherapy is usually given after a lumpectomy when a patient was diagnosed with DCIS or IDC [4]. However, radiochemoresistance is a common barrier for survival rate improvement [6, 7, 9, 25]. Having demonstrated that miR-671-5p inhibits EMT, which is implicated in the development of BC therapeutic resistance [26], we next addressed whether miR-671-5p reverses therapeutic resistance by inhibiting FOXM1-mediated EMT and/or DNA repair function. To observe the dynamic effects of miR-671-5p on radiotherapy during the BC progression, we treated miR-671-5p-transfected 21T cells with UV exposure or chemotherapeutic agents in indicated doses after 24 h and 48 h following miR-transfection. The sensitivity was determined by the MTT assay. As shown in Fig. 6a, miR-671-5p overexpression either significantly or partially increased cell sensitivity to UV and cisplatin, paclitaxel, and epirubicin, but not to 5-Fu in 21T cell lines compared to mock-transfected cells. Inhibition of miR-671-5p resulted in an inverse effect. Additionally, we performed rescue experiments to further validate whether FOXM1 was involved in miR-671-5p-mediated sensitization of T21 cells to UVC exposure and chemotherapeutic agents. Re-expression of FOXM1 with pcDNA3.1/FOXM1 plasmid in 21T cell lines reduced cell sensitivity to UV and chemotherapeutic agents in all 21T cells (Additional file 2: Figure S2A). These data suggest that miR-671-5p may be a new therapeutic target for DCIS and IDC as it reverses radiochemoresistance through inhibition of FOXM1.

### miR-671-5p reduced DNA repair capability in 21T cell lines

**Fig. 7:**
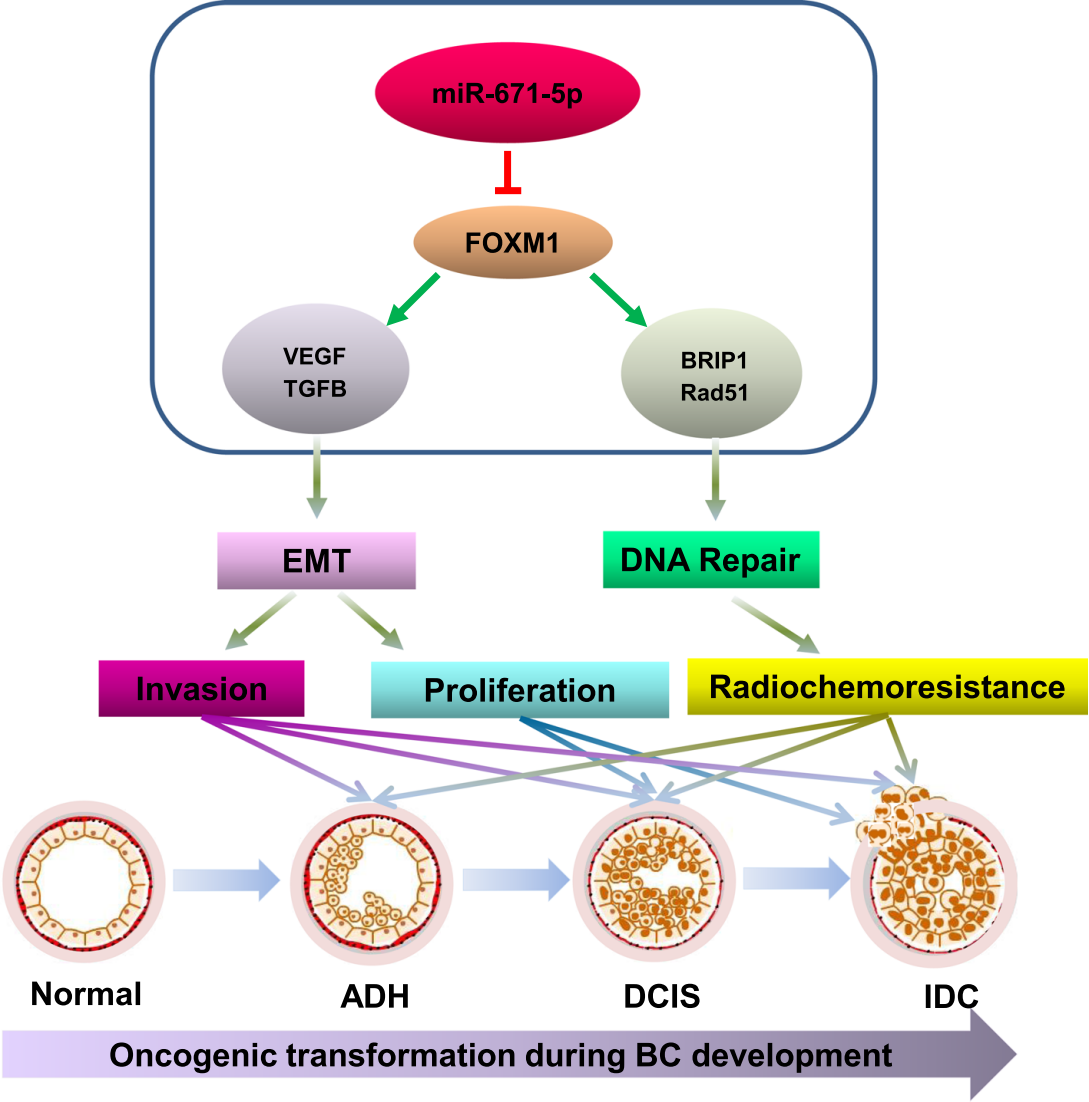
A schematic model for the dynamic regulation of miR-671-5p during the BC oncogenic transformation. miR-671-5p directly targets FOXM1. Downregulation of FOXM1 could (1) inhibit EMT-associated cell proliferation in DCIS (21NT) and IDC (21MT) cell lines, (2) inhibit EMT-associated invasion in ADH (21PT), DCIS (21NT), and IDC (21MT) cell lines, and (3) improve DNA repair capability-associated UV sensitivity in all stages during the oncogenic transformation of BC

Alteration in DNA repair efficiency has been considered one of the critical factors involved in radiochemoresistance [27]. Consistent with an important role of FOXM1 in DNA repair [12], our prior work demonstrated that miR-671-5p decreased DNA repair capability by targeting FOXM1, leading to increased sensitivity to radiotherapy and chemotherapy in MDA-MB-231 cells [19]. To further determine the dynamic effects of miR-671-5p on radiochemoresistance during the progression of BC, we quantitatively assessed DNA damage and repair capability of miR-671-5p to UV and chemotherapeutic agents by HCR assays. pCMU-Luc vector pre-treated by UVC and chemotherapeutic agents was co-transfected with miR-671-5p into 21T cells and the luciferase activity was measured. Cells co-transfected with UV pre-damaged pCMU-Luc and miR-671-5p exhibited significantly reduced post-UV HCR activity compared to the mock-co-transfected one in 21MT cell lines. The slightly reduced post-UV HCR activity was still observed in H16N2, 21PT, and 21NT, despite the difference did not reach statistical significance. In contrast, co-transfection of pre-damaged pCMU-Luc with miR-671-5p inhibitor inversed the post-UV HCR activity in all 21T cells compared to the inhibitor mock one (Fig. 6b). The post-chemo HCR was examined by co-transfection of pCMU-Luc pre-damaged by chemotherapeutic agents with miR-671-5p. Co-transfection of cisplatin pre-damaged pCMU-Luc with miR-671-5p exhibited significantly reduced post-cis HCR activity compared to the mock-co-transfected control in H16N2, 21NT, and 21MT. Surprisingly, significantly increased post-cis HCR activity was observed in 21PT cells, suggesting ADH might be a specific stage in which other factors may play a role in miR-671-5p-mediated FOXM1 inhibition. Co-transfection of cisplatin pre-damaged pCMU-Luc with miR-671-5p inhibitor rescued the luciferase activity in all 21T cell lines. Interestingly, although miR-671-5p did not affect 5-Fu sensitivity in 21T cells (Fig. 6a), we still observed suppression of miR-671-5p on post-5Fu HCR in H16N2, 21TPT, and 21NT cell lines (Fig. 6b), suggesting that miR-671-5p-mediated FOXM1 inhibition does not affect 5-Fu resistance. For post-paclitaxel and epirubicin HCR, we found significantly or partially reduced DNA repair capability when co-transfection of paclitaxel or epirubicin pre-damaged pCMU-Luc with miR-671-5p in 21PT, 21NT, and 21MT, which is also consistent with the effect of miR-671-5p in paclitaxel or epirubicin drug resistance. Rescue experiment was performed by co-transfection of pre-damaged pCMU-Luc with miR-671-5p or mock plus pcDNA3.1-FOXM1 or pcDNA3.1 empty plasmid. These data showed that re-expression of FOXM1 abrogated the alleviating effect of miR-671-5p on DNA repair compared to the pcDNA3.1 empty plasmid one (Fig. 7b). Taken together, we demonstrated that at least partially, miR-671-5p plays an important role in reducing DNA damage by targeting FOXM1, which improves the sensitivity to chemo-radiation therapy for patients with both earlier and later stage BC.

### miR-671-5p suppressed the expression of FOXM1 downstream genes involving in EMT and DNA repair pathways

Having demonstrated that miR-671-5p represses EMT and DNA repair by targeting FOXM1 during BC progression, we asked if the inhibition of miR-671-5p on FOXM1 expression affects the downstream gene(s) involved in EMT and DNA repair during BC progression. EMT program is mediated by complex signaling networks induced by different factors. We analyzed the expression of these genes when miR-671-5p was overexpressed. As expected, overexpression of miR-671-5p resulted in significant downregulation of TGF-β and VEGF in 21NT and 21MT (Additional file 1: Figure S1). FOXM1 has been reported as a transforming growth factor-beta (TGF-β) inducer and enhanced TGF-β-induced EMT [28, 29]. In addition, FOXM1 is a transcriptional factor of vascular endothelial growth factor (VEGF) during EMT [30–32]. These results suggest that miR-671-5p may serve as a key repressor of FOXM1-mediated EMT.

FOXM1 is an essential regulator of DNA damage response and genotoxic agent resistance [12]. We hypothesized that miR-671-5p sensitize 21T cells to radio-chemo treatment by targeting FOXM1 and modulating FOXM1 downstream genes involved in DNA repair. Overexpression of miR-671-5p significantly reduced the expression of two downstream genes of FOXM1, BRIP1 (BRCA1 interacting protein C-terminal helicase 1), a DNA repair protein that works with BRCA1 to repair damaged DNA [33], and RAD51 (RAD51 Recombinase), a protein which assists in the repair of DNA double-strand breaks [34] (Additional file 3: Figure S3). These results suggest that miR-671-5p sensitizes radiochemotherapy by targeting FOXM1-mediated DNA repair proteins, BRIP1 and RAD51.

## Discussion

The current model of human BC progression proposes a linear multistep process which initiates as ADH, evolves into DCIS, and culminates in the potentially lethal stage of IDC that requires a dynamic accumulation of molecular alterations. Prompt detection and intervention of early BC and/or precancerous lesions improves the patients’ survival rate. Our data showed a gradual, dynamic decreased expression of miR-671-5p and its tumor suppressor function during the oncogenesis of human BC in the 21T series cell lines that mimics specific stages of human BC progression. In clinical samples, the expression of miR-671-5p displays a gradually dynamic decrease from ADH, to DCIS, to IDC in both FFPE tissues and serums. Statistically significant correlation was seen between the histological grade and miR-671-5p down-expression in FFPE tissues. To determine whether the dynamic change of miR-671-5p can be used as a circulating biomarker, we also analyzed miR-671-5p expression in serum from individuals diagnosed with benign breast lesion, ADH, DCIS, and IDC. Consistent with the results from FFPE tissues, we observed a gradually decreased miR-671-5p expression from benign breast lesions, to ADHs, DCISs, and IDC (Fig. 1b). Although the gradually decreased miR-671-5p expression was not statistically significant among the different steps due to their small numbers, it provides a clue for the application of circulating miR-671-5p expression in BC diagnosis. More importantly, we found that the expression levels of miR-671-5p can differentiate between cADH and sADH. The functional analysis revealed that miR-671-5p inhibits proliferation in advanced stages and invasion in early stages in 21T cell lines. The observation in which miR-671-5p is able to enhance cell proliferation more significantly in advanced stages may be explained by the presence of high endogenous miR-671-5p levels in H16N2 and 21PT cell lines, which reduces the efficiency of forced ectopic miR-671-5p by transfection. Our results suggest that decreased miR-671-5p is an important risk factor in BC progression and may serve as a biomarker in both FFPE tissue and serum for early detection.

The mechanism underlying decreased miR-671-5p during BC progression is not yet fully understood. miRNA expression is associated with complex multilevel regulations such as somatic copy-number alterations (SCNAs), transcriptional and post-transcriptional mechanisms, and the effects of endogenous (hormones, cytokines) and exogenous (xenobiotics) compounds in relation to cell types, physiological states of the body, and various external factors [35]. Further investigation is needed to explore genomic and epigenetic alterations in miR-671-5p regulation.

FOXM1 is associated with EMT phenotype [36] and DNA damage repair [12]. miR-671-5p appears to suppress EMT and sensitize to UV and chemotherapeutic (except 5-Fu) in 21NT and 21MT cell lines more significantly than in 21PT and H16N2 cell lines via the suppression of FOXM1. To the best of our knowledge, this is the first study to show a linear tumor-suppressive role of miR-671-5p in BC progression, which might serve as a biomarker for early detection and prognosis (Fig. 7).

In this study, we found that miR-671-5p significantly reversed the resistance to UV, cisplatin, and paclitaxel treatment (albeit slightly to epirubicin) and that such findings are consistent with the predicted function of miR-671-5p in DNA repair modulation (Fig. 6). Although FOXM1 has been associated with 5-Fu resistance, we did not observe any effect of miR-671-5p on resistance to 5-Fu in 21T cell lines. Nevertheless, we still observed a slightly reduced DNA repair capability to 5-Fu damaged DNA after miR-671-5p transfection (Fig. 6). This may be due to the fact that in addition to the DNA repair pathway, the resistance to 5-Fu is conferred by additional mechanisms. For example, increased thymidylate synthase and dihydropyrimidine dehydrogenase (DPD) are major molecular mechanism responsible for 5-FU resistance [37].

## Conclusions

We expanded our previous work to assess the tumor suppressor function of miR-671-5p during the oncogenesis of BC. Loss of miR-671-5p leads to the activation of FOXM1-mediated EMT progression and enhanced DNA repair capability, resulting in a gradually aggressive molecular event in the development of radio/chemoresistance (Fig. 7). The present study raises the possibility that miR-671-5p may be a potential biomarker for BC early detection and therapy.

## Additional files

Additional file 1: Figure S1. Transfection of miR-671-5p inhibitor abrogated the effect of miR-671-5p on cell morphology. (PNG 144 kb)

Additional file 2: Figure S2. Transfection of FOXM1 abrogated the effect of miR-671-5p on radiochemotherapy and DNA repair capability. (PNG 162 kb)

Additional file 3: Figure S3. miR-671-5p suppressed the expression of FOXM1 downstream genes involving EMT and DNA repair pathway. (PNG 139 kb)

## Data Availability

All data generated or analyzed during this study are included in this published article.

## Abbreviations

3′UTR: 3′-Untranslated region
ADH: Atypical ductal hyperplasia
BC: Breast cancer
DCIS: Ductal carcinoma in situ
EMT: Epithelial-to-mesenchymal transition
FOXM1: Forkhead box protein M1
HCR: Host cell reactivation
IDC: Invasive ductal carcinoma
TNBC: Triple-negative breast cancer
