# Supplementary figures and images for: Dynamically decreased miR-671-5p expression is associated with oncogenic transformation and radiochemoresistance in breast cancer
Source: Breast Cancer Res. 2019 Aug 7;21:89. doi: 10.1186/s13058-019-1173-5 (PMC6686561; doi:10.1186/s13058-019-1173-5)

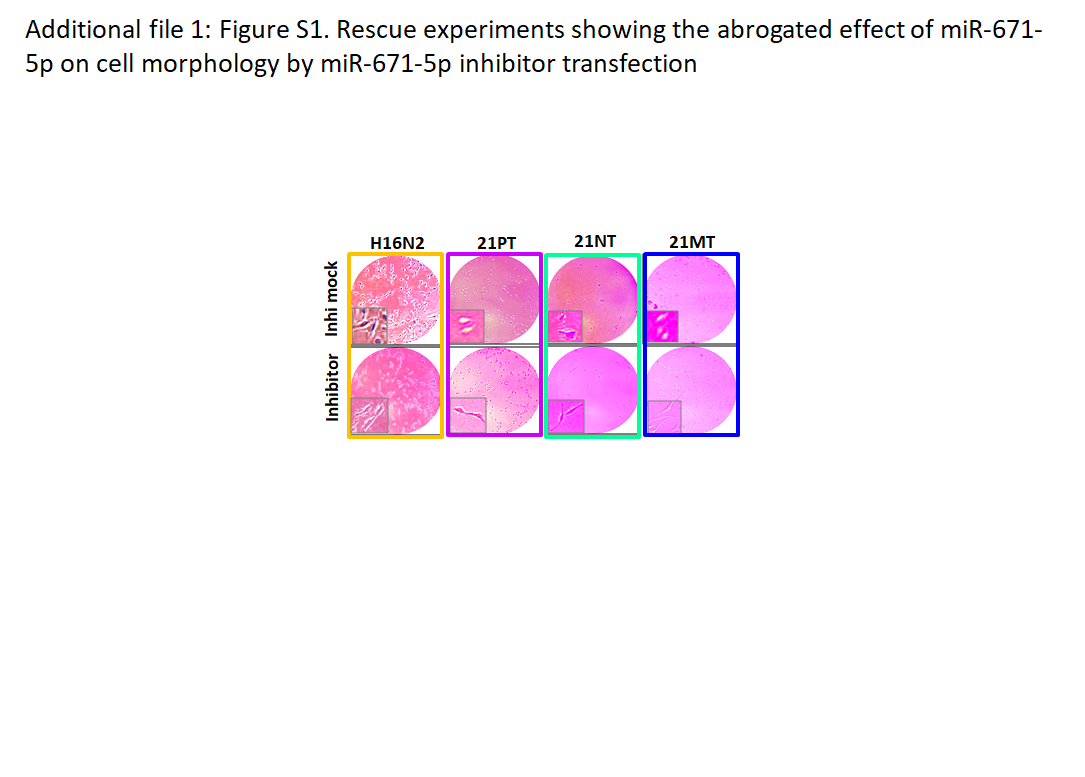

Supplement: Supplementary file 1 — Figure S1. Transfection of miR-671-5p inhibitor abrogated the effect of miR-671-5p on cell morphology. (PNG 144 kb) [file 13058_2019_1173_MOESM1_ESM.png]

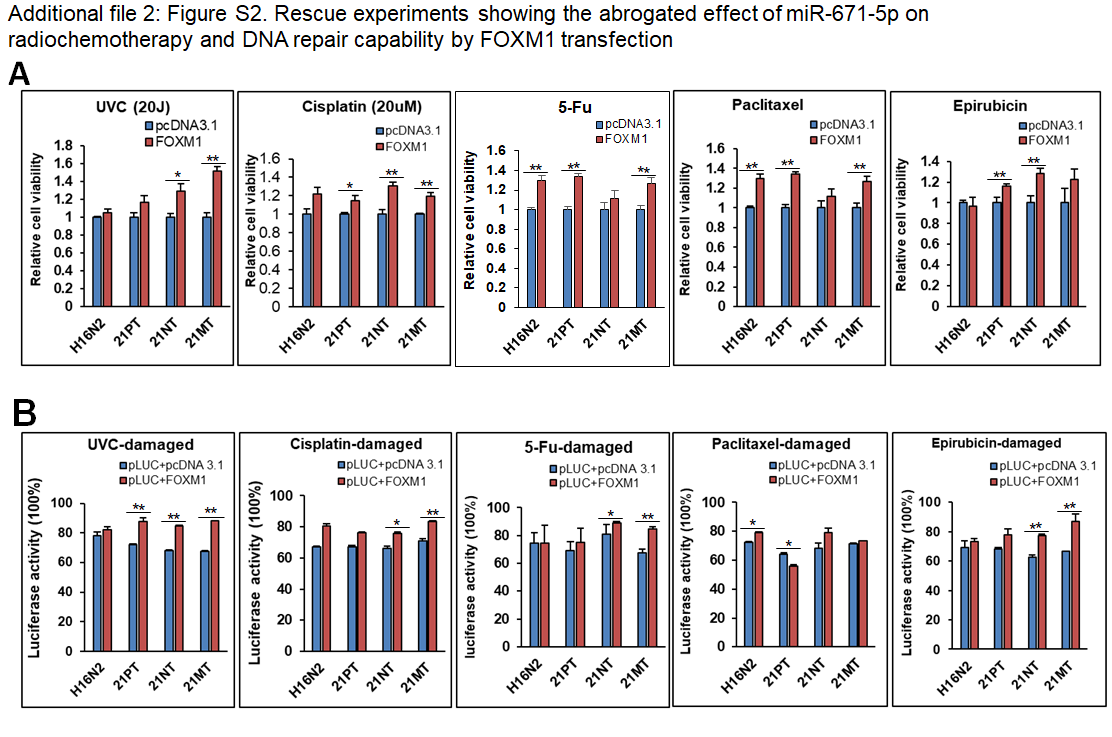

Supplement: Supplementary file 2 — Figure S2. Transfection of FOXM1 abrogated the effect of miR-671-5p on radiochemotherapy and DNA repair capability. (PNG 162 kb) [file 13058_2019_1173_MOESM2_ESM.png]

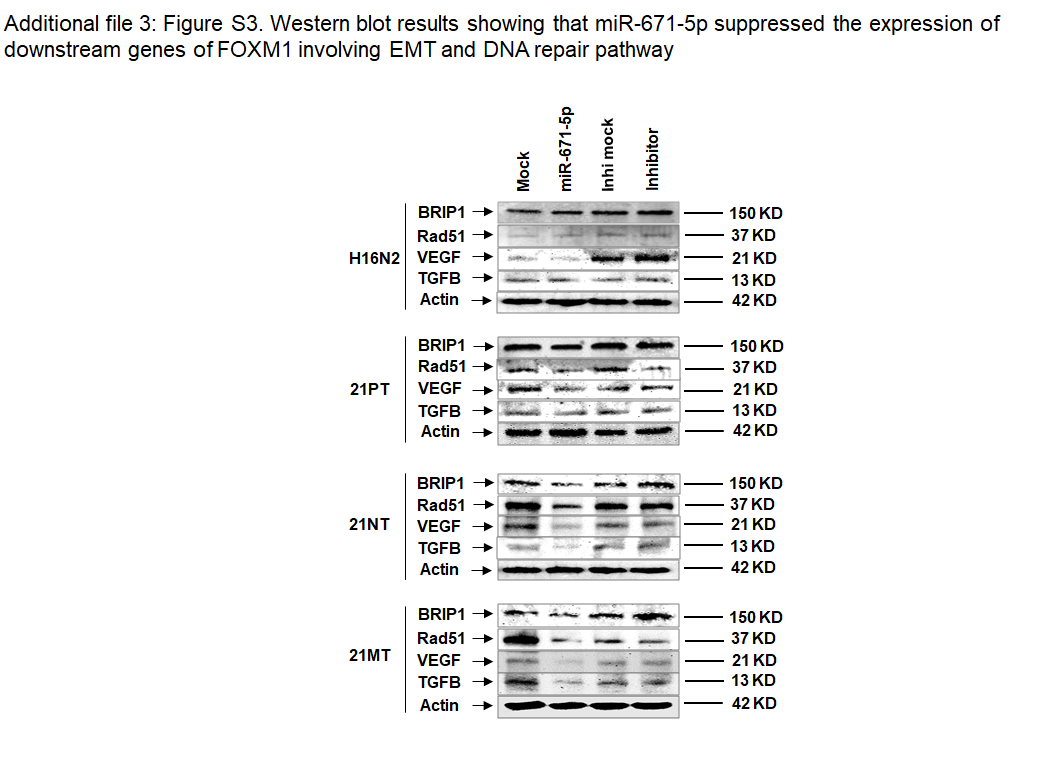

Supplement: Supplementary file 3 — Figure S3. miR-671-5p suppressed the expression of FOXM1 downstream genes involving EMT and DNA repair pathway. (PNG 139 kb) [file 13058_2019_1173_MOESM3_ESM.png]
